# Molecular Mechanisms Underlying the Circadian Rhythm of Blood Pressure in Normotensive Subjects

**DOI:** 10.1007/s11906-020-01063-z

**Published:** 2020-07-13

**Authors:** Yves Lecarpentier, Olivier Schussler, Jean-Louis Hébert, Alexandre Vallée

**Affiliations:** 1 Centre de Recherche Clinique, Grand Hôpital de l’Est Francilien, 77104 Meaux, France; 2 Department of Thoracic surgery, Nouvel Hôpital Civil, Hôpitaux Universitaires de Strasbourg, Strasbourg, France; 3 Department of Cardiovascular Surgery, Research Laboratory, Geneva University Hospital, Geneva, Switzerland; 4 Cardiology Institute, Pitié-Salpétrière Hospital, AP-HP, Paris, France; 5 Diagnosis and Therapeutic Center, Hypertension and Cardiovascular Prevention Unit, Paris-Descartes University, Hôtel-Dieu Hospital, AP-HP, Paris, France

**Keywords:** Arterial blood pressure, Canonical WNT/β-catenin pathway, Glycogen synthase kinase, Astrocyte, Circadian rhythm, Suprachiasmatic nucleus, Glutamine synthetase, nucleus tractus solitarius

## Abstract

**Purpose of Review:**

Blood pressure (BP) follows a circadian rhythm (CR) in normotensive subjects. BP increases in the morning and decreases at night. This review aims at providing an up-to-date overview regarding the molecular mechanisms underlying the circadian regulation of BP.

**Recent Findings:**

The suprachiasmatic nucleus (SCN) is the regulatory center for CRs. In SCN astrocytes, the phosphorylated glycogen synthase kinase-3β (pGSK-3β) also follows a CR and its expression reaches a maximum in the morning and decreases at night. pGSK-3β induces the β-catenin migration to the nucleus. During the daytime, the nuclear β-catenin increases the expression of the glutamate excitatory amino acid transporter 2 (EAAT2) and glutamine synthetase (GS). In SCN, EAAT2 removes glutamate from the synaptic cleft of glutamatergic neurons and transfers it to the astrocyte cytoplasm where GS converts glutamate into glutamine. Thus, glutamate decreases in the synaptic cleft. This decreases the stimulation of the glutamate receptors AMPA-R and NMDA-R located on glutamatergic post-synaptic neurons. Consequently, activation of NTS is decreased and BP increases. The opposite occurs at night.

**Summary:**

Despite several studies resulting from animal studies, the circadian regulation of BP appears largely controlled in normotensive subjects by the canonical WNT/β-catenin pathway involving the SCN, astrocytes, and glutamatergic neurons.

## Introduction

Numerous physiological regulations vary rhythmically over the course of a 24-h day. Circadian rhythms (CRs) such as body temperature, sleep-wake cycles, metabolism, and blood pressure (BP) represent various examples of CRs. Although BP is relatively stable over time in normotensive subjects [1], it varies throughout the day, according to a CR [2–4]. Thus, BP starts to rise during the morning (morning surge), about 1 h prior to awakening and decreases at night [5]. Once the BP peak is reached, it remains elevated until late afternoon and starts to progressively decrease to reach a nadir around 3:00 (a 10–20% dip). CRs in the brain, heart, kidneys, and arterial vessels prepare the transition from sleep to activity and adjust the cardiovascular system for optimal function [6]. An abnormal CR of the BP is considered to be an excellent predictor of adverse cardiac events [7–13]. Rhythmic variations in BP require great interest as several cardiovascular diseases, such as myocardial infarction, stroke, arrhythmia, and sudden cardiac death, are linked to CR dysregulations [14–16]. These events often occur in patients whose BP fails to decline during the night. Such patients are called non-dippers [13, 17]. In mammals, the central clock resides in the suprachiasmatic nucleus (SCN) which is directly stimulated by light throughout the retinal hypothalamic tract (RHT). Peripheral clocks located in most other tissues throughout the body, are synchronized by the SCN in response to light and communicate via either neuronal or hormonal signals [18–20]. The disruption of normal day-night cycles, such as those induced by jet lag, leads to desynchronization between the central SCN clock and peripheral clocks [21]. BP follows a CR, independently of the sleep/wake cycle or fasting/feeding [22]. If physical activity is increased during the night, the nocturnal decrease in BP is blunted. Thus, there is a link between the CR of BP and physical activity, showing that the sleep/awake ratio of BP is related to the night/day activity [7]. Shift works alter CRs. Shift workers, i.e., subjects working beyond the typical daily working hours, night shift, early morning shift, and rotational work have a higher chance of hypertension than day workers do [23]. The longer the workers work continuously, the higher the risk of hypertension.

In human normotensive subjects, the cellular and molecular mechanisms underlying the CR of arterial BP are not well understood and remain to be determined. This review focuses on the key role of the phosphorylation of the glycogen synthase kinase-3β (pGSK-3β), a major component of the canonical WNT/β-catenin pathway. The β-catenin-induced expression of the glutamate excitatory amino acid transporter 2 (EAAT2) and glutamine synthetase (GS) modulates the glutamate cycle [24•]. Importantly, pGSK-3β follows a CR in SCN astrocytes [25•, 26•] that ultimately regulates the sympathetic/parasympathetic nervous activity and induces a circadian regulation of the arterial BP. Circadian variations of pGSK-3β in combination with those of EAAT2 and GS via the canonical WNT/β-catenin signaling, but also with those of other pathways such as those of PI3K-Akt [27–29] and cMYC [30], contribute to a circadian modulation of the glutamate content in both the cytoplasm of astrocytes and in the synaptic cleft of the glutamatergic neurons in the SCN. Finally, the re-uptake of glutamate by EAAT2 in the morning induces a reduction in its content in the synaptic cleft. This leads to a decrease in the activation of the nucleus tractus solitarius (NTS) and therefore to an increase in BP during the day. The opposite occurs at night.

## The Core Clock Genes

CRs are biological processes displaying endogenous oscillations of about 24 h. They are driven by a number of important clock genes. The core clock genes consist of BMAL1 (brain and muscle aryl-hydrocarbon receptor nuclear translocator-like 1), CLOCK (Circadian Locomotor Output Cycles Kaput), PER (period), and CRY (cryptochrome) [31]. They are closely regulated and form interlocking feedback and feed-forward loops. In the nucleus, BMAL1 and CLOCK proteins form a heterodimer that binds to E-box elements in their promoter regions. This activates the PER and CRY transcription. After accumulation in the cytoplasm, PER and CRY proteins heterodimerize and then translocate to the nucleus where they repress the CLOCK-BMAL1 regulatory complex, thereby inhibiting their own transcription. From the cytoplasm, the nuclear orphan receptors RORα and REV-ERBα translocate to the nucleus. RORα activates and REV-ERBα represses ROR-mediated transcription, forming the secondary autoregulatory feedback loops. REV-ERBα is activated by CLOCK-BMAL1 and feeds back to transcriptionally repress BMAL1. The activator clock genes (CLOCK and BMAL1) are expressed in anti-phase to the suppressor clock genes (PERs and CRYs). Duration of the circadian clock cycle can be regulated by post-translational events that include phosphorylation by kinases [32]. This whole complex system regulates the circadian rhythmicity of numerous biochemical pathways [31]. The central clock together with peripheral clocks within the kidneys, brain, heart, and vasculature play an important role in the circadian regulation of BP [33, 34].

## The Master Circadian Clock Suprachiasmatic Nucleus

**Fig. 1:**
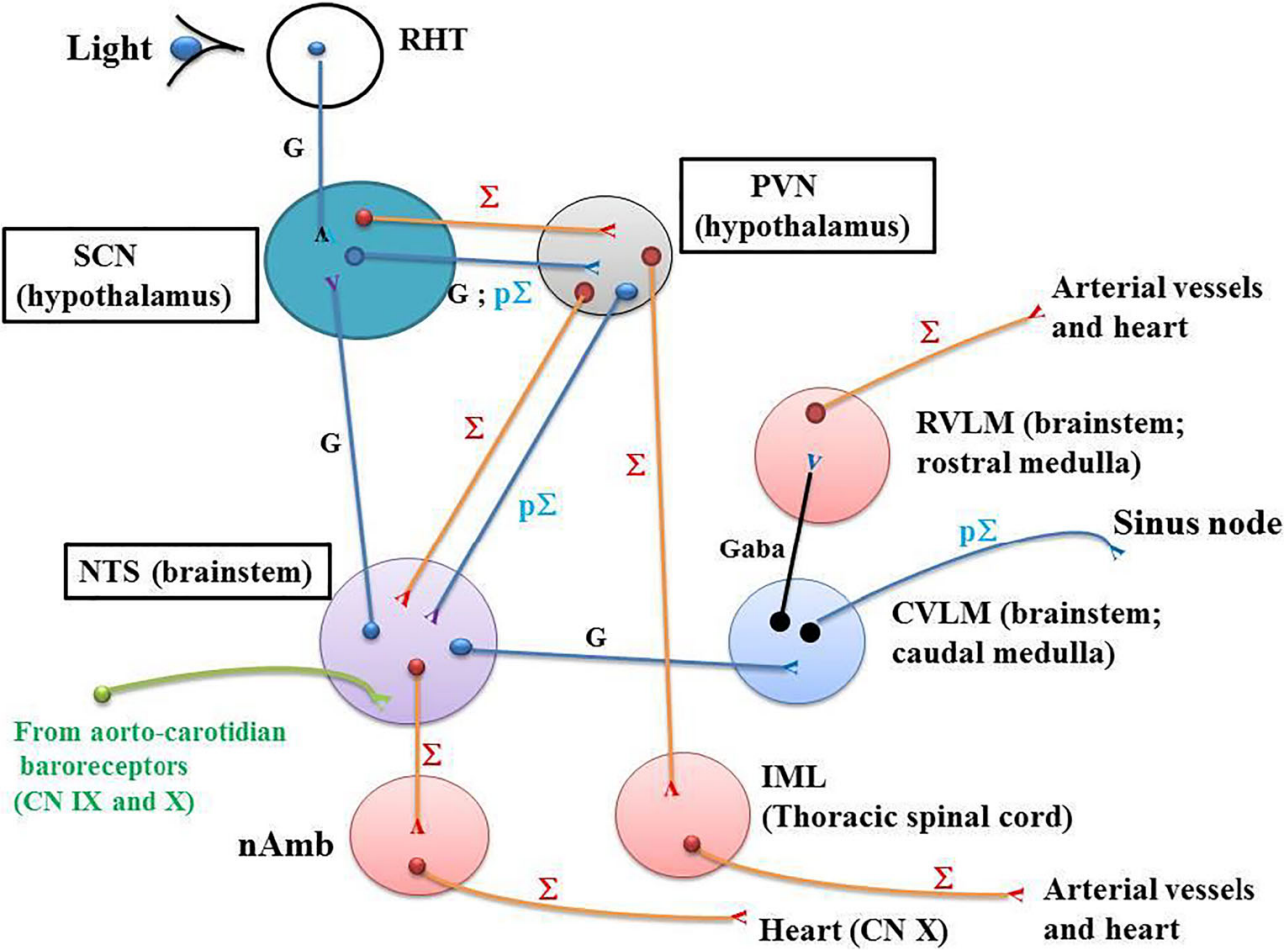
Neuroanatomical network involved in the CR of BP. RHT: the retinohypothalamic tract (RHT) originates in the intrinsically photosensitive retinal ganglion cells (ipRGC) which project directly via monosynaptic and glutamatergic transmission to the ventrolateral SCN through the optic nerve and the optic chiasma. ipRGC relay photic information from the eyes to the SCN. SCN: the suprachiasmatic nucleus (SCN) receives glutamatergic neurons from both RHT and nucleus tractus solitarius (NTS). SCN projects glutamatergic pre-autonomic parasympathetic neurons to the PVN. Separate pre-autonomic sympathetic or parasympathetic neurons project to pre-autonomic neurons of the PVN. PVN: paraventricular nucleus (PVN) receives pre-autonomic sympathetic and parasympathetic neurons from the SCN. PVN projects pre-ganglionic sympathetic neurons in the IML. Moreover, pre-autonomic sympathetic neurons in the PVN present axon collaterals to pre-autonomic parasympathetic neurons in the PVN itself and to the NTS [47•, 55]. NTS: nucleus tractus solitarius (NTS) which is located in the brainstem receives neuronal informations from the aortic and carotid baroreceptors which are stimulated after a rapid increase in BP. The carotid sinus baroreceptor axons travel within the glossopharyngeal nerve. The aortic arch baroreceptor axons travel within the vagal nerve. Baroreceptor activity travels along these nerves directly to the NTS. Then, the neuronal activity flows from NTS to both parasympathetic and sympathetic neurons within the brainstem. The NTS neurons send glutamatergic excitatory fibers to the caudal ventrolateral medulla (CVLM). The GABAergic activated CVLM then sends inhibitory fibers to the rostral ventrolateral medulla (RVLM), thus inhibiting the RVLM. The sympathetic and parasympathetic branches of the autonomic nervous system have opposite effects on BP. When the aortic and carotid baroreceptors are activated by an increase in BP, NTS activates CVLM, which in turn inhibits the RVLM. This decreases the activity of the sympathetic branch of the autonomic nervous system, leading to a decrease in BP. Conversely, a decrease in BP decreases baroreceptor activation and induces an increase in sympathetic tone via “disinhibition” of the RVLM. CVLM: caudal ventrolateral medulla (CVLM)*.* The ventrolateral medulla consists of the caudal ventrolateral medulla (CVLM) and the rostral ventrolateral medulla (RVLM)*.* The ventrolateral medulla, part of the medulla oblongata of the brainstem, plays a major role in regulating arterial BP. CVLM receives neurons from the NTS and projects GABA fibers towards RVLM, which in turn inhibits the activity of the RVLM. RVLM: rostral ventrolateral medulla (RVLM). Neurons in the RVLM project directly to pre-ganglionic neurons in the spinal cord (intermediolateral nucleus: IML) which maintain tonic activity of the sympathetic vasomotor nerves. The RVLM is the primary regulator of the sympathetic nervous system, sending glutamatergic excitatory fibers to the sympathetic pre-ganglionic neurons located in the IML of the spinal cord. IML: the intermediolateral nucleus (IML) is located in the lateral grey column of the spinal cord and mediates the entire sympathetic innervation of the body. It receives neurons from the RVLM. nAmb: the nucleus ambiguus (nAmb) is located in the medullary reticular formation and gives rise to efferent motor fibers of the vagal nerve (CN X). These fibers are cardio-inhibitory allowing rapid BP changes in response to fast increases in BP. The parasympathetic outflow arising from nAmb acts to decrease cardiac activity in response to fast increases in BP. nAmb receives fibers from NTS. Symbols: Σ; sympathetic (red); pΣ: parasympathetic (blue); G: glutamatergic neurons

In mammals, SCN located in the hypothalamus is the master circadian clock that coordinates daily rhythms of behavior and metabolism [18]. SCN (Fig. 1) interacts with peripheral oscillators, drives, and synchronizes the daily activity of CRs to the light/dark cycle [19] via transcriptional-translational feedback loops resulting in rhythmic expression of the core clock genes. SCN synchronizes rhythms of clock gene expression in all body organs. Thus, the body is prepared in an optimal manner and adapted to daily changes in activity. The electrical activity of numerous SCN neurons presents CRs that induce a circadian secretion of neurotransmitters towards target areas of the body [35–37]. The SCN also imposes its own rhythm into the body through the autonomous nervous system. The SCN uses separate connections for the sympathetic and parasympathetic systems. Dysfunction of the SCN initiates cardiovascular diseases, particularly in several types of hypertension [38–40]. Postmortem analyses have reported abnormalities in the SCN of hypertensive humans and animals [41, 39, 42].

## Neuroanatomical Centers

### The Retinal Hypothalamic Tract

The ventrolateral SCN [43] is involved through light inputs coming from the retina via the RHT (Fig. 1). In mammals, RHT plays a key role in the regulation of CRs. RHT originates in the photosensitive retinal ganglion cells (ipRGC). The ipRGC axons of the RHT project directly to the SCN by means of a monosynaptic junction to the SCN through the optic nerve and the optic chiasma. They relay photic information from the eyes to the SCN. Light-induced information enters the ventrolateral SCN. Light activation of RHT makes it possible for CRs to adjust to the bright or dark environment. SCN receives and interprets information according to the level of environmental light. Retinal nerve terminals contain a significant concentration of glutamate. Light acts as a zeitgeber, which is an external or environmental cue that synchronizes biological rhythms to the Earth’s 24-h light/dark cycle, 12-month cycle, etc. Light thus induces a powerful effect on the SCN. Glutamate, a key neurotransmitter, is stored in a subpopulation of ipRGC projecting to the retinal-recipient zone of the ventrolateral SCN. Glutamate mediates light signaling to the SCN. Glutamate and glutamate agonists such as N-methyl-d-aspartate mimic light-induced phase shifts. A glutamate immune reactivity within presynaptic nerve terminals has been reported in the rat SCN [44, 45]. In the SCN, the targets of glutamate are the clock genes PER1 and PER2, which are influenced by light [46].

### The Suprachiasmatic Nucleus

The ventrolateral SCN receives direct glutamatergic projections from the caudal nucleus tractus solitaries (NTS) controlling the cardiovascular areas (Fig. 1). This provides a key neuroanatomical link between NTS and SCN for the involvement of the ventrolateral SCN in the short-term control of BP. These neurons are glutamatergic ones [47^•^]. This has been corroborated by experiments in which either NTS or SCN is lesioned. Thus, a BP rise induced by an α1-agonist infusion in animals leads to an increase in neuronal activity in both caudal NTS and ventrolateral SCN. Lesioning in the caudal NTS prevents this neuronal activity [47^•^]. Other studies have also shown that damages of the caudal NTS drastically impair the short-term adaptation of BP after administration of a vasoactive agent [48, 49]. Importantly, the BP rise induced by a vasopressive stimulus increases to more than twice the BP level observed in SCN-lesioned animals [47•]. This demonstrates the role of the SCN in counteracting a rise in BP and in the short-term regulation of BP. Thus, the NTS-SCN neuroanatomical connection (Fig. 1) damps down the BP increase after a vasopressive stimulus. Moreover, SCN lesions alter the 24-h BP variability in rats [50]. The ventrolateral SCN adapts its response according to the BP information that originates in the caudal NTS. The SCN receives feedback on the cardiovascular status via the NTS. The NTS is itself the major integrative site towards which a large amount of peripheral information converges from both aortic and carotid baroreceptors (Fig. 1). Activation of the ventrolateral SCN through caudal NTS neuronal projections stimulated by an increase in BP or through light-RHT-SCN activation leads to a similar pattern [51]. Moreover, SCN projects pre-autonomic sympathetic and parasympathetic neurons to the paraventricular nucleus (PVN) [47•]. SCN balances separate pre-autonomic sympathetic and parasympathetic neurons to peripheral organs [52]. Thus, the ventrolateral SCN provides a quadruple function: (i) SCN coordinates the regulation of CRs [20]. SCN synchronizes the peripheral clock genes involved in the cardiovascular control, comprising arterial blood vessels, kidneys, and adrenal glands [53, 54]; (ii) SCN damps down a BP increase via the complex NTS-SCN-PVN neuroanatomical network [47]; (iii) SCN is activated by light via RHT glutamatergic neurons [51]; (iv) SCN exports glutamatergic neurons to the PVN.

### The Paraventricular Nucleus

PVN exports pre-autonomic sympathetic neurons to NTS and the intermedio-lateralis nucleus (IML). Pre-autonomic sympathetic neurons in PVN have collaterals that project to pre-autonomic parasympathetic neurons in the NTS [52, 55, 47•] (Fig. 1). There are reciprocal connections between neurons in the NTS and the PVN. This shows that the PVN is involved in the neural control of the cardiovascular system [56]. Certain neuronal projections from the PVN to the NTS are glutamatergic [57].

### The Nucleus Tractus Solitarius (NTS)

The NTS, located in the brainstem (Fig. 1) is central in the short-term BP regulation and represents a key element of the baroreflex loop. The most sensitive baroreceptors are located in both the carotid sinuses and the aortic arch. The baroreceptor activity travels along the glossopharyngeal nerve and vagal nerve up to the NTS. NTS neurons send glutamatergic excitatory fibers to the caudal ventrolateral medulla (CVLM). The activated CVLM then sends inhibitory GABAergic fibers to the rostral ventrolateral medulla (RVLM) which is the primary regulator of the sympathetic nervous system. RVLM sends excitatory fibers to the sympathetic pre-ganglionic neurons located in the IML of the spinal cord (Fig. 1). An increase in BP activates baroreceptors which results in stimulating the activity of neurons in the caudal NTS [58, 59]. Then, NTS activates the CVLM via glutamatergic fibers which increases the activity of the parasympathetic branch of the autonomic nervous system, and in turn inhibits the RVLM, thus decreasing the activity of the sympathetic branch of the autonomic nervous system. This leads to a decrease in BP. Conversely, a decrease in BP deactivates baroreceptors leading to an increase in sympathetic tone via “disinhibition” of the RVLM and an increase in BP.

### Light-Induced Effects on the SCN

The environmental light which is provided by the day-night cycle impacts on CRs through both RHT and SCN, controlling the homeostasis of blood glucose level, water balance, and body temperature [20, 60, 61]. Importantly, the autonomic nervous activity is modified after light exposure, an effect that disappears after SCN lesioning [62, 63]. Light, through RHT and SCN, enhances sympathetic activity and suppresses vagal outflows, whereas SCN lesions eliminate these changes [20]. The autonomic nervous system contributes to the CR of BP [64, 65]. However, connections between light-induced molecular events and neurophysiological excitability have not been definitively identified [66]. GSK-3β appears to play a key role in the regulation of mammalian CRs and particularly in the phosphorylation of several components of the core-molecular clock [26•, 67, 68]. Chronic pGSK-3β activation increases SCN neuronal activity, whereas inhibition of pGSK-3β diminishes it, highlighting pGSK-3β as a regulator of neurophysiological CRs [69, 70]. Importantly, in the SCN, GSK-3β regulates the photic signaling [71]. In the SCN, we have seen earlier that pGSK-3β exhibits a CR with maximum activity in the morning at ZT2. It then progressively decreases during the daytime, until it reaches a minimum at night ZT14, after which it begins to increase again [26•]. Light is an important entraining signal to the SCN [72, 73]. At specific times in the night, exposure to light shifts the phase of the molecular clock and behavioral CR [73]. In response to an acute light-pulse, the phase-resetting mechanism induces a glutamate release from the RHT onto SCN neurons [74, 75, 46] and activation of NMDA receptors [76, 77]. In mice SCN, a late-night exposure to light significantly increases the GSK-3β activity (corresponding to a decreased pGSK-3β level), which occurs 30–60 min after a light-pulse [71]. Thus, GSK-3β activation mediates light-induced SCN excitability and photic entrainment [71]. Conversely, inhibition of GSK-3β blocks the light-induced excitability in SCN neurons. Moreover, GSK-3β activation is necessary for light-induced SCN neuronal activity [71]. These results are consistent with those from other studies that have shown a light-induced decrease in the number of pGSK3β positive cells in rat SCN [78]. GSK-3β regulates CRs in SCN excitability through the modulation of a persistent sodium current (INaP) [70]. Optogenetic stimulation of SCN neurons alone is sufficient to shift CRs, mimicking the effects of a light-pulse [79]. GSK-3β dysregulation has been found to be associated with chronic night lightning exposure and to be linked with several disorders such as depression [80], obesity [81, 82], and cancer [83].

### GSK-3β in SCN Astrocytes

**Fig. 2:**
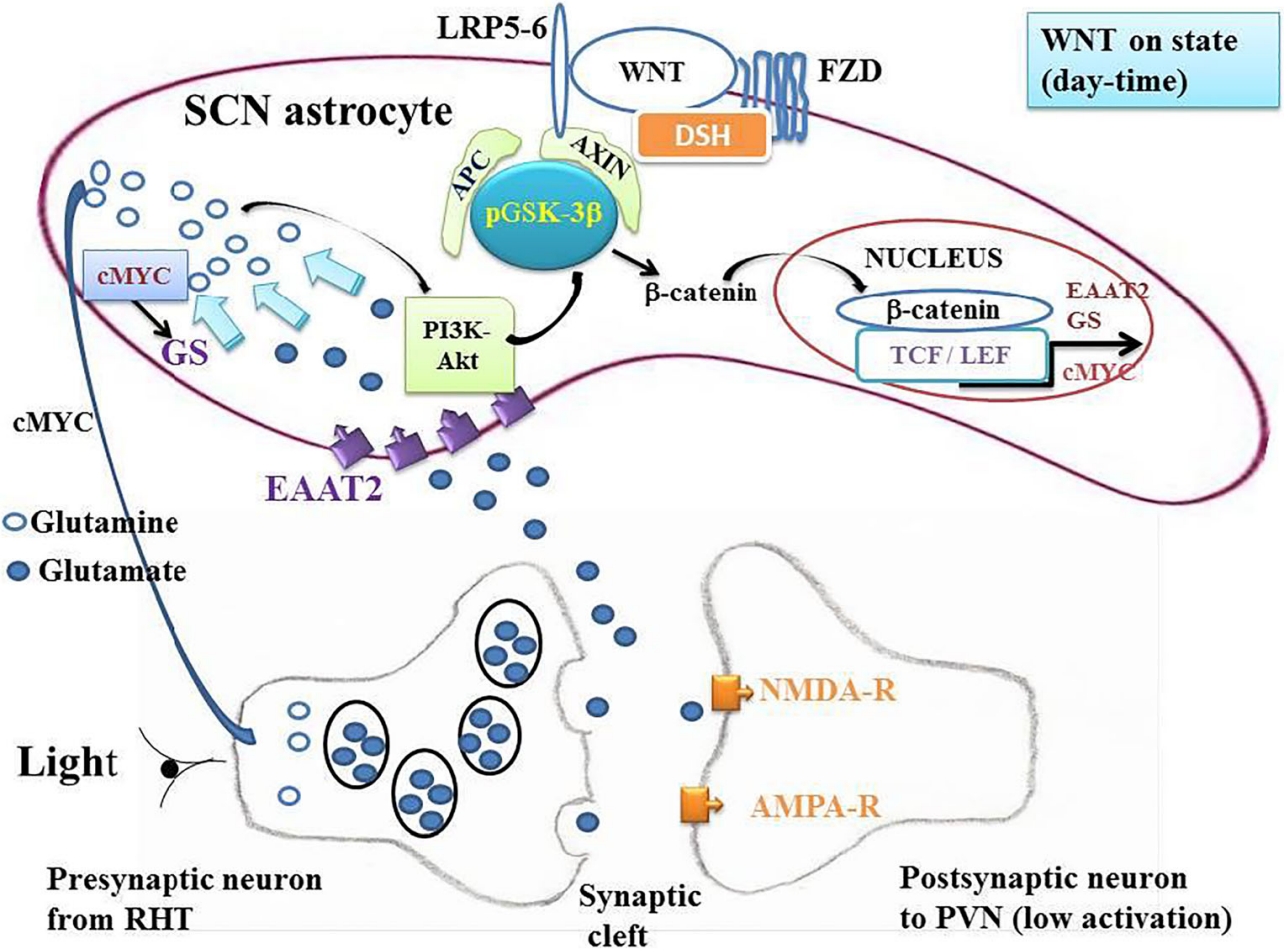
Phosphorylated glycogen synthase kinase-3β (pGSK-3β) and canonical WNT/β-catenin signaling in “on” state. The canonical WNT pathway is “on-state” in the presence of the inactive phosphorylated form of the glycogen synthase kinase-3β (pGSK-3β). Phosphorylation of GSK-3β can be induced either by WNT ligands or by other molecular mechanisms and particularly by light. On the one hand, a WNT ligand binds both FZD and LRP5/6 receptors. GSK-3β is then under the inactive phosphorylated form pGSK-3β. This leads to activation of the phosphoprotein Disheveled (DSH). DSH recruits the destruction complex (pGSK-3β + AXIN +APC) to the plasma membrane, where AXIN directly binds the cytoplasmic tail of LRP5/6. APC is the adenomatous polyposis coli. On the other hand, expression of pGSK-3β follows a circadian rhythm and reaches a maximum in the morning at ZT2. In both mechanisms, β-catenin is detached from the destruction complex, accumulates into the cytosol, and then translocates to the nucleus to bind the LEF-TCF co-transcription factors. This induces the WNT-response gene transcription of numerous genes such as EAAT2, GS, and cMYC. This favors the re-uptake of glutamate from the synaptic cleft to the cytoplasm of astrocytes. This leads to a decrease of glutamate content in the synaptic cleft and a decrease of the activation of NMDA-R and AMPA-R, which are receptors located in the synaptic part of the glutamatergic post-synaptic neuron. SCN neurons project to the NTS via PVN and consequently decrease the parasympathetic tone and increase the sympathetic tone which favors the increase in BP in the morning when the expression of pGSK-3β is increased. β-Catenin increases the expression of cMYC. cMYC stimulates GS. Glutamine in the SCM astrocyte cytoplasm stimulates the PI3K-Akt pathway. The PI3K-Akt pathway phosphorylates GSK-3β

**Fig. 3:**
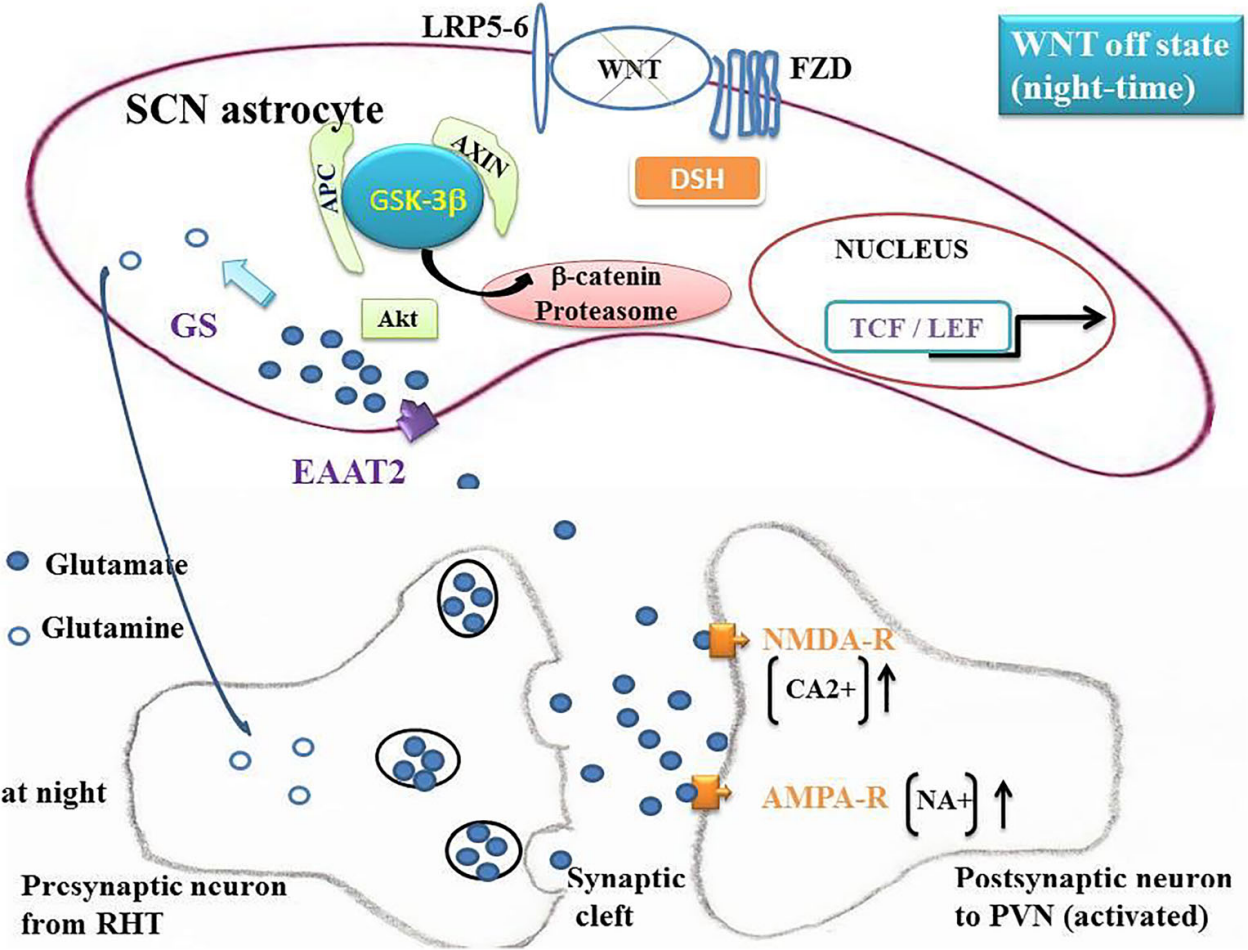
Unphosphorylated glycogen synthase kinase-3β (GSK-3β) and canonical WNT/β-catenin signaling in “off” state. Due to the decrease in the phosphorylation of pGSK-3β at night, the unphosphorylated form GSK-3β increases. This occurs at night reaching a nadir at ZT14. The canonical WNT pathway becomes in “off-state.” The cytosolic β-catenin is phosphorylated and undergoes the destruction process into the proteasome. This decreases the expression of EAAT2, GS, and cMYC. Thus, the re-uptake of glutamate into the SCN astrocyte cytoplasm decreases and the glutamate content increases in the synaptic cleft, leading to an augmentation of NMDA-R and AMPA-R activation. This leads to an increase in parasympathetic tone and a diminution of the sympathetic tone and *in fine* a decrease in BP at night.

pGSK-3β is a major component of the destruction complex in the canonical WNT/β-catenin pathway [84–87] (Figs. 2 and 3). The phosphorylated form (pGSK-3β) activates this pathway and the non-phosphorylated form (GSK-3β) inhibits it. In SCN astrocytes, pGSK-3β allows the release of β-catenin from the destruction complex. Cytoplasmic β-catenin then migrates from the cytoplasm to the nucleus of SCN astrocytes. The nuclear β-catenin links the co-transcription factors TCF/LEF. This leads to an increase in the expression of both EAAT2 and GS in astrocytes [24•]. This makes it possible the transport of glutamate from the synaptic cleft to the cytoplasm of SCN astrocytes and, consequently, this lowers the glutamate content in the synaptic cleft thereby decreasing activation of NMDA-R and AMPA-R located in the post-synaptic glutamatergic neuron, the stimulation of which is diminished (Figs. 2 and 3).

The two GSK-3 isoforms, i.e., GSK-3α and GSK-3β, are inactivated by phosphorylation at Ser-21 for GSK-3α (S21-GSK-3α) and Ser-9 for GSK-3β (S9-GSK-3β). Importantly, in the SCN, both phosphorylated GSK-3α (pGSK-3α) and phosphorylated GSK-3β (pGSK-3β) isoforms show circadian variations. The phosphorylation state of S9-GSK-3β changes through the light-dark cycle [25•, 26•]. In the SCN, the pGSK-3β isoform (inactive state) presents a CR [25•, 26•, 88]. Circadian changes in total pGSK-3β content are undetectable, but importantly, the phosphorylation level at Ser-9 (S9-GSK-3β) changes in a circadian manner in both SCN and liver and can be measured [26•]. This suggests that the rhythmic phosphorylation of pGSK-3β at Ser-9 is regulated by the SCN master clock center, while the liver peripheral clock is regulated via a systemic control from the SCN. The significant CR of both phosphorylated GSK-3α and GSK-3β has also been observed in the SCN from wild-type mice housed in constant darkness for 2 weeks [88]. The persistence of the CR of pGSK-3β in the absence of light argues for an intrinsic clock regulation. GSK-3 directly phosphorylates at least five core clock proteins, i.e., CLOCK, BMAL1, PER2, CRY2, and REV-ERBα [89, 90, 67, 68]. BMAL1 undergoes various post-translational modifications and is specifically phosphorylated by GSK-3β on Ser 17 and Thr 21. In the absence of GSK-3β-mediated phosphorylation, BMAL1 becomes stabilized and its gene expression is dampened. This makes it possible to control the stability of BMAL1 as well as the amplitude of the CR. BMAL1 phosphorylation is a key regulatory mechanism in maintaining the robustness of the CR [68].

### Astrocytes Regulate CRs in SCN [91–93]

β-Catenin signaling positively regulates glutamate uptake in SCN astrocytes [24•]. The glutamate/glutamine cycle plays a major role in the brain homeostasis. Glutamate is the most abundant neurotransmitter in the brain and is involved in learning, memory, and cognition. The regulation of the glutamate cycle involves two major elements: (i) glutamate transporter EAAT2 in humans and glutamate transporter GLT-1 in rodents, and (ii) GS. Glutamate is released from the presynaptic part of the neuron towards the synaptic cleft. The astrocyte membrane contains EAAT2 transporters which are assigned to clear glutamate from the synaptic cleft towards the astrocyte cytoplasm (Figs. 2 and 3). Then, glutamate is converted into glutamine by GS that is exclusively expressed in astrocytes [94] and that catalyzes the ATP-dependent reaction of glutamate and ammonia into glutamine. Glutamine is released from astrocytes and in turn is taken up by the presynaptic neuron to be converted back to glutamate by a glutaminase. Astrocytes play a major role in the excitatory pathway and clear more than 90% of glutamate excess [95, 96]. EAAT2 is primarily expressed on astrocytes, and is the major transporter of glutamate into astrocytes [97, 98].

### EAAT2 and GS in SCN Astrocytes

β-Catenin is a transcriptional co-activator and a key mediator of the canonical WNT/β-catenin pathway (Figs. 2 and 3). It positively regulates EAAT2 and GS at the transcriptional level in human progenitor–derived astrocytes (PDAs) by partnering with T cell factors 1 and 3 (TCF-1 and TCF-3) [24•]. β-Catenin knockdown results in a decrease in the EAAT2 and GS expression in the prefrontal cortex. EAAT2 and GS are responsible for excitatory glutamate neurotransmission and the glutamate cycle in vitro and in vivo. Disruption of the canonical WNT/β-catenin signaling leads to the modification of at least 128 genes [99]. Both in vitro and in vivo, disruption of β-catenin induces a dramatic inhibition of the glutamate transport network (EAAT2/GS) within astrocytes.

A modified WNT/β-catenin pathway together with neuroinflammation has been described in aged rats and HIV patients [100, 101]. An abnormal β-catenin pathway impacts the EAAT2/GS expression and ultimately induces a glutamate excitotoxicity that represents a common neuropathological pathway for neuroinflammation and neurodegeneration. Anomalies in β-catenin regulation of both EAAT2 and GS have been reported in mouse hepatocellular carcinoma and human pancreatic tumors [102–104]. Numerous dysfunctions in the glutamate cycle have been reported. Thus, impairment of the EAAT2 function has been observed in acquired immune deficiency syndrome, amyotrophic lateral sclerosis, Alzheimer’s disease, epilepsy, and ischemia/stroke [101, 105–117]. An excess of glutamate is neurotoxic and leads to numerous neurodegenerative diseases such as Alzheimer’s disease [118, 119] and Parkinson’s disease [120, 121].

### Phosphorylation of GSK-3β

The PI3K-Akt pathway activates the phosphorylation of GSK-3β. Akt is activated by the phosphatidylinositol 3-kinase (PI3K) signaling. Akt inhibits the active form GSK3-β and activates the inactive form pGSK-3β [29]. As previously seen, this makes it possible the release of β-catenin in the astrocyte cytoplasm and its translocation in the nucleus leading to the β-catenin-induced expression of EAAT2 and GS. In the brain, glutamate induces a rapid, reversible, and dose- and time-dependent loss of Akt-1 phosphorylation and kinase activity [27, 122].

Early in the morning and during the day, pGSK-3β activates the β-catenin signaling. This requires that the PI3K-Akt pathway is activated, since PI3K-Akt phosphorylates GSK-3β and that the glutamate content is low, as glutamate inhibits the PI3K-Akt pathway [27] and consequently inhibits pGSK-3β. Since the expression of both EAAT2 and GS is increased during the daytime period, it is necessary that the transformation of glutamate into glutamine by GS is greater than or equal to the glutamate uptake by EAAT2. Moreover, β-catenin increases the expression of GS but also that of cMYC which itself also activates GS [30]. A relatively low glutamate level is required in the cytoplasm of SCN astrocytes. Moreover, glutamine activates PI3K-Akt [28], which perennializes activation of the canonical WNT signaling during the day. This pathway decreases or reverses during the night until ZT14 when pGSG-3β reaches a nadir. The glutamate content in astrocytes depends on the relative interaction between the EAAT2 activity which tends to increase glutamate content in astrocytes and the GS activity which tends to diminish it. At ZT2, pGGK-3β is maximum and the expression of EAAT2 increases [26•]. Consequently, the re-uptake of glutamate in the astrocyte cytoplasm increases, whereas glutamate in the synaptic cleft decreases, leading to lowering of the activation of glutamate receptors (AMPA-R and NMDA-R). Thus, activation is decreased in the pre-autonomic parasympathetic neurons projecting from the SCN to the PVN. This results in a decrease in the NTS activation, leading to a decrease in the parasympathetic (CVLM) tone and an increase in the sympathetic tone (RVLM), thus leading in an increase in BP during the daylight period. The opposite happens at night.

### A Model of the Circadian Rhythm of BP in Normotensive Subject (Fig. 4)

**Fig. 4:**
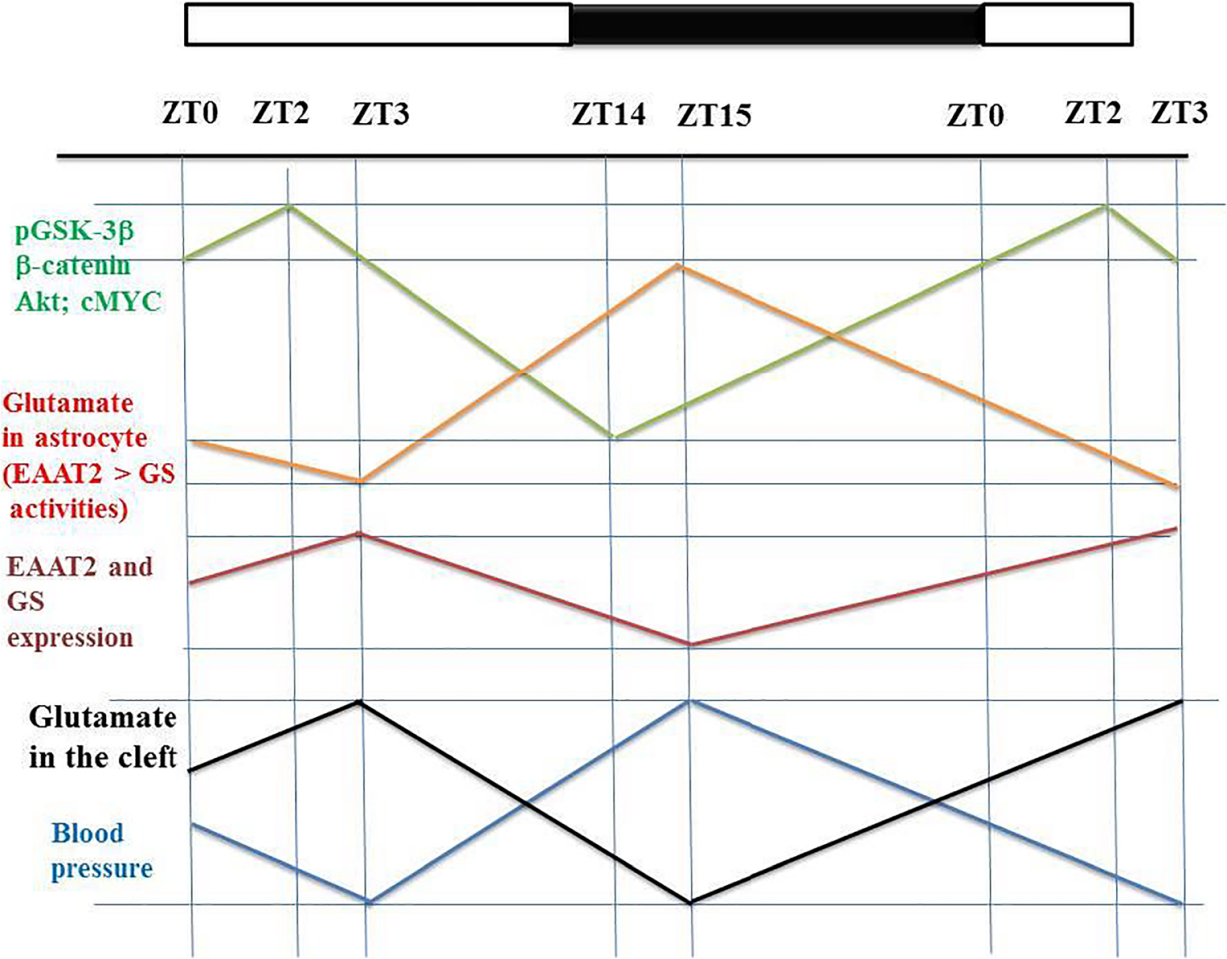
Simplified model of the CR of the BP. pGSK-3β, Akt activation, and nuclear β-catenin vary in a parallel manner and decrease from ZT2 to ZT14. Glutamate astrocyte content is the difference between EAAT2 activity and GS activation. Glutamate content in astrocyte cytoplasm inhibits the Akt pathway and consequently the GSK-3β phosphorylation which progressively decreases from ZT2 to ZT14. EAAT2 and GS vary in parallel with pGSK-3β, but with a short delay. Glutamate content in the synaptic cleft activates the SCN glutamatergic neurons projecting to the PVN and subsequently to the NTS. This increases the parasympathetic tone and inhibits the sympathetic tone, leading to decrease BP during the night. The glutamate content in the cleft progressively decreases from ZT3 to to ZT15, which decreases the parasympathetic tone and increases the sympathetic tone, resulting in an increase in BP during the daytime

GS catalyzes glutamate into glutamine and glutamine promotes the activity of the protein kinase Akt in vivo [28] (Figs. 2 and 3). Thus, an increase in glutamine content in the astrocyte cytoplasm activates PIK3-Akt and consequently favors the phosphorylation of GSK-3β- and the β-catenin-dependent expression of both EAAT2 and GS. Moreover, the WNT/β-catenin signaling stimulates c-MYC expression [123]. It is also important to note that cMYC stimulates the expression of GS [30]. Thus, GS activity is promoted by the β-catenin-dependent increased expression of both GS and c-MYC. cMYC activates GS activity and glutamine overproduction activates Akt activity. During the daytime, the high GS activity in the astrocyte cytoplasm makes it possible to maintain a low cytoplasmic glutamate content, a high glutamine content, and a high level of Akt-dependent level of phosphorylated GSK-3β. GSK-3β phosphorylation, Akt activation, cMYC activation, and nuclear β-catenin levels vary in a parallel manner and decrease from ZT2 to ZT14 (Fig. 4). Glutamate content in astrocyte is the difference between the EAAT2 uptake capacity and the GS activation. High glutamate content in the astrocyte cytoplasm inhibits the PI3K-Akt pathway and consequently the GSK-3β phosphorylation which decreases from ZT2 to ZT14. EAAT2 and GS vary parallel to pGSK-3β with a short delay (Fig. 4). Glutamate content in the synaptic cleft activates the SCN glutamatergic post-synaptic neurons projecting to the PVN, and subsequently to the NTS. This favors the parasympathetic tone and inhibits the sympathetic tone, leading to a decrease in BP at night. The glutamate content in the synaptic cleft decreases from ZT3 to ZT15, which decreases the parasympathetic tone and increases the sympathetic tone, leading to an increase in BP during the daytime (Fig. 4).

### Thermodynamics and BP

BP follows a CR and from a thermodynamic point of view, CRs are dissipative structures. This term has been first used by Ilya Prigogine [124] to characterize complex structures created by irreversible processes occurring far from equilibrium. Such conditions can produce oscillations and can drive certain systems to organized states [125]. Irreversible processes generate entropy, which is commonly thought to be disorder. Surprisingly, the same irreversible processes can also produce self-organization, and dissipative structures [126, 127]. These concepts apply to temporal and sustained oscillations [128–130]. This makes it possible to bring together within a common formalism the great variety of rhythms and particularly CRs, reported at different levels of the biological organization, independently of numerous underlying mechanisms. CRs control major physiological functions, and their dysfunction can lead to numerous pathological states. They predict when sunlight will return, and this makes it possible the appropriate orchestration of the entire physiological machinery before sunrise. This leads to a competitive advantage. In terms of thermodynamic, this can modify the total entropy production rate compared to systems that lack such anticipatory control. Relating the entropy production rate to irreversible processes is of major importance. In the modern thermodynamics formulation, the change in entropy dS is the sum of two parts: dS = d_e_S + d_i_S, (d_i_S ≥ 0), in which d_e_S is the entropy change due to exchange of matter and energy with the exterior of the system and d_i_S is the entropy change due to irreversible processes within the system. Irreversible processes can be expressed in terms of thermodynamic flows and thermodynamic forces. The entropy production rate due to all the irreversible processes is the product of a thermodynamic force (*F*) and a thermodynamic flow (*J*), leading to the equation d_i_S / dt = *F* × *J* [125, 131]. In mammal hemodynamics, the arterial blood pressure (BP), which is a controlled variable, is the product of the thermodynamic force *R* (peripheral arterial resistances) and the thermodynamic flow *Q* (cardiac output). Thus, d_i_S/dt = BP = Q × R. The decrease in BP overnight leads to a decrease in the rate of entropy production and therefore is accompanied by a decrease in irreversible processes. This represents a considerable thermodynamic advantage in animal species where BP follows a circadian rhythm. The disappearance of the circadian regulation of BP amplifies the genesis of irreversible processes and promotes the occurrence of many pathologies in humans.

## Conclusions

Although numerous studies cited in this review have been carried out in animals, we observe that the canonical WNT/β-catenin pathway plays a central role in accounting for the circadian variation of BP in the normotensive subject. The crucial factor explaining the CR of BP is the circadian regulation of pGSK-3β in SCN astrocytes. Light stimulates the sympathetic nervous system and inhibits the parasympathetic nervous system through the SCN. SCN astrocytes generate a complex mechanism in which the canonical WNT/β-catenin pathway participates through the phosphorylation of GSK-3β and thanks to the neuroanatomical RHT-SCN-PVN-NTS axis. EAAT2 increases the glutamate re-uptake from the synaptic cleft to the astrocyte cytoplasm. This decreases activation of the SCN and NTS during the day resulting in an increase in BP. The opposite occurs at night. Phosphorylation of GSK-3β reaches a maximum at ZT2. The concomitant increase in the expression of EAAT2 leads to a decrease in glutamate level in the synaptic cleft. This deactivates the AMPA-R and NMDA-R receptors as well as the post-synaptic glutamatergic neurons in both the SCN and NTS. This induces an increase in BP during the day. The circadian phosphorylation of GSK-3β via the canonical WNT/β-catenin signaling induces the expression of EAAT2 and GS in astrocytes. This makes it possible the increase of BP during the day and consequently the increase in the entropy production rate. At night, BP and the entropy production rate both decrease, minimizing irreversible processes. The circadian BP regulation constitutes a dissipative structure that operates far from equilibrium. From a thermodynamic point of view, the presence of a CR of BP confers a favorable status. Entropy production rate decreases at night, which minimizes the production of irreversible processes during the nocturnal period of the nychthemeron. The canonical WNT/β-catenin pathway plays a major role in mediating hypertensive heart disease [132]. Disruption of any element of this cascade may help to explain the occurrence of hypertension.
